# Middle Aortic Syndrome in a Child-Bearing Age Patient

**DOI:** 10.1055/s-0039-1688933

**Published:** 2019-10-15

**Authors:** Andrew P. Rabenstein, Khaled F. Salhab, Georgios Spentzouris, Vijayapraveena Paruchuri, George Hines, Anthony M. Vintzileos, Scott L. Schubach

**Affiliations:** 1 Department of Surgery, Stony Brook University Hospital, Stony Brook University, Stony Brook, New York; 2 Department of Cardiothoracic Surgery, NYU Winthrop University Hospital, Mineola, New York; 3 Department of Vascular Surgery, NYU Winthrop University Hospital, Mineola, New York; 4 Department of Cardiology, NYU Winthrop University Hospital, Mineola, New York; 5 Department of Obstetrics and Gynecology, NYU Winthrop University Hospital, Mineola, New York

**Keywords:** aorta, thoracoabdominal aorta, pregnancy

## Abstract

We report a rare case of a 30-year-old female who had a long-standing history of middle aortic syndrome that was being managed nonsurgically. She presented with hypertension and buttock pain with plans to become pregnant. She underwent an aortoiliac bypass.

## Introduction

Middle aortic syndrome consists of segmental narrowing of the aorta typically involving the lower thoracic and upper abdominal aorta with or without renal artery involvement. This results in arterial hypertension and symptoms of chronic lack of blood flow. It is usually secondary to either a congenital developmental anomaly of the aorta or to one of many acquired conditions such as Takayasu's arteritis, fibromuscular dysplasia, neurofibromatosis, or mucopolysaccharidosis. 1 It is a very rare condition, comprising 0.5 to 2.0% of aortic coarctations. 2 If left untreated, it leads to life-threatening complications secondary to severe hypertension, with significant mortality at a young age. Treatment usually entails aortoaortic bypass of the diseased segment through a thoracoabdominal approach, with excellent prognosis in the majority of patients. We present the case of a young female with symptomatic middle aortic syndrome with the intention of getting pregnant in the near future.

## Case Presentation

A 30-year-old female with middle aortic syndrome, diagnosed at the age of 15, presented to us with hypertension and significant buttock numbness after a brief period of sitting down. Past cardiac history was also remarkable for a bicuspid aortic valve and mild mitral regurgitation. She was medically managed with an angiotensin-converting enzyme inhibitor and β-blockers for hypertension and was a nonsmoker who expressed strong wishes to become pregnant. Her obstetrician was concerned that her condition would not support her pregnancy owing to lack of blood flow to the pelvis during pregnancy. The patient had two prior spontaneous miscarriages. Physical examination was remarkable for hypertension, with systolic blood pressure ranging from 160 to 180 mm Hg. Strong palpable pulses were noted in her upper extremities, with weaker palpable pulses in her lower extremities.

**Fig. 1:**
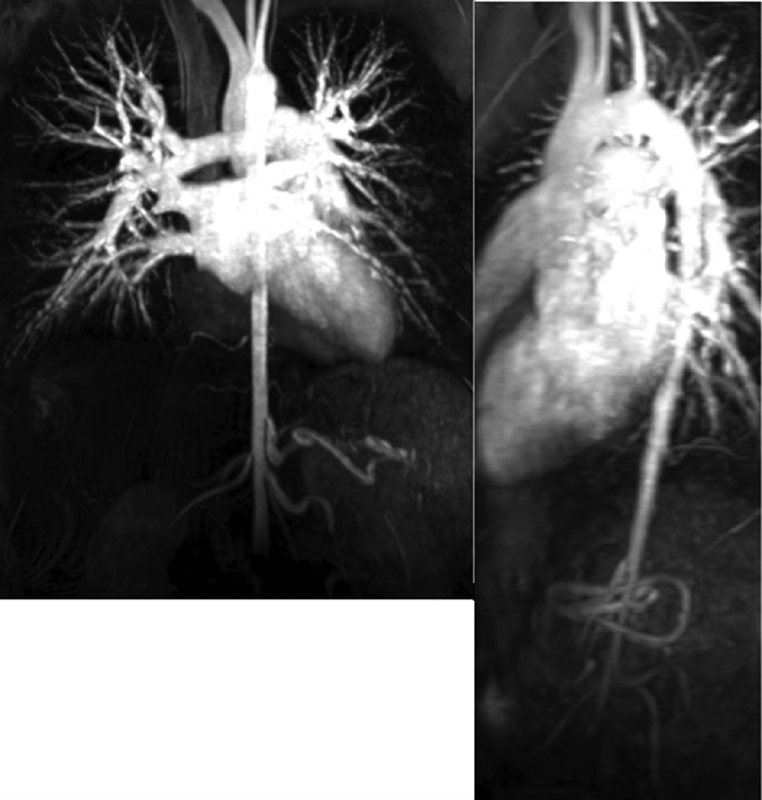
Magnetic resonance angiogram imaging of the patient's aorta preoperatively.

Preoperative work-up included a magnetic resonance (MR) angiogram which revealed a normal appearing thoracic aorta that tapers from 2.0 cm down to 8 mm in size. There was no evidence of stenosis at the origin of the renal arteries or in the mesenteric vessels ( Fig. 1 ). A dynamic cardiac MR revealed a maximal gradient of 37 mm Hg at the level of the diaphragm.

The patient underwent a tunneled descending thoracic aorto-left common iliac artery bypass, avoiding the need for a traditional large thoracoabdominal incision. The thoracic aorta was accessed via a 10-cm left thoracotomy incision and the left common iliac via an 8-cm left paramedian incision. A retroperitoneal tunnel was then created, and an opening in the diaphragm was made to enter the thoracic cavity. The bypass was performed using a 22 cm × 9 mm Dacron graft between the descending thoracic aorta and the proximal left common iliac artery tunneled through the neo-diaphragmatic hiatus. She did very well in recovery and was discharged from the hospital on postoperative day 5 without any complications.

At her 3-week follow-up visit, the patient reported complete resolution of her buttock pain and was able to discontinue her antihypertensive medication. The patient subsequently became pregnant and delivered a full-term baby. At 1.5 years' follow-up, the patient continues to do well.

## Discussion

Middle aortic syndrome is an extremely rare cause of arterial hypertension, resulting from an acquired or congenital condition causing segmental narrowing of the descending aorta. If left untreated, the majority of patients usually die because of progressive severe hypertension before the age of 35 to 40. The term “middle aortic syndrome” was first coined by Sen et al 3 who described in 1962 four cases of narrowing of the aorta at unusual sites in the subisthmal aorta. Taketani et al 4 described their experience of surgical treatment of atypical aortic coarctation complicating Takayasu's arteritis in 33 cases over 44 years. The aortic coarctation was proximal to the origin of the renal arteries in 29 patients with hypertension. In the vast majority of cases, treatment was surgical with an aortoaortic bypass using a 10- to 16-mm prosthetic graft via a left thoracoabdominal incision.

Ours is an unusual case where an aorto-left common iliac bypass was performed to treat symptoms, increase survival, and prevent potential maternal and fetal morbidity during pregnancy via a minimally invasive approach through a tunnel through the diaphragm. To our knowledge, this has not been previously reported.

Pregnancy is associated with several significant physiologic changes in the cardiovascular system, including systemic vasodilation that occurs as early as 5 weeks of gestation. There is also up to 45% increase in cardiac output, and an increase in total blood volume by approximately 45% that is proportionally more than red blood cell mass, resulting in physiologic anemia. 5 These changes are necessary to adapt for the increased metabolic demands of both the mother and the fetus. Cardiovascular disease in pregnancy is the leading cause of maternal mortality in North America. 6 Some call pregnancy a “nature's stress test” since it can unmask underlying cardiovascular pathology. It has been well documented that pregnant patients with Takayasu's arteritis, an idiopathic, nonspecific aortoarteritis that can potentially cause segmental aortic narrowing, have worse fetal outcome with significantly higher perinatal mortality compared with unaffected patients. 7 8

Our patient had managed her aortic coarctation nonsurgically as long as possible. The standard repair involves a traditional thoracoabdominal incision. Endovascular interventions involving balloon angioplasty and/or stenting may also be an option, but they are limited to isolated aortic stenoses without visceral involvement, and the long-term durability is unknown. 9 10 In a recent retrospective study, surgical bypass was compared with endovascular treatment for patients with supra-aortic arterial occlusive disease in Takayasu's arteritis. 11 In that series, surgical bypass demonstrated better patency rates compared to endovascular treatment. In addition, symptom recurrence was more common with an endovascular approach. 11

Our goal was to decrease expected mortality, ensure adequate uteroplacental circulation, and prevent fetal demise. The underlying condition that our patient had, which included an average 8-mm abdominal aortic diameter, coupled with her strong desire to have a successful first child, made the situation highly risky.
